# A collaborative immunohistochemical study of Drp1 and cortactin in the epithelial dysplasia and oral squamous cell carcinoma

**DOI:** 10.1186/s13000-025-01627-0

**Published:** 2025-04-11

**Authors:** Marina Nader, Samar Soliman, Shaimaa M. Yussif, Azza Abbas El-Sissi

**Affiliations:** 1 Faculty of Dentistry, Mansoura University, Mansoura, Egypt; 2 Faculty of Medicine, Mansoura University, Mansoura, Egypt

**Keywords:** OSCC, IHC, Drp1, Cortactin

## Abstract

**Objectives:**

Oral squamous cell carcinoma (OSCC) accounts for more than 90% of oral malignancies. The poorly understood molecular and cellular mechanisms underlying the pathogenesis of OSCC remain a subject of paramount importance. For epithelial dysplasia, invasion, and metastasis to occur, tumor cells require energy obtained from the mitochondria and phenotypic cellular changes in the actin cytoskeleton. Dynamin-related protein1 (Drp1) is one of the main mitochondrial proteins regulating the mitochondrial dynamics. Cortactin is an actin-binding protein that promotes the actin polymerization and rearrangement. The interplay between both proteins in OSCC remains elusive. The current study aimed to investigate the immunohistochemical (IHC) expression of Drp1 and cortactin in tissues revealing propagating OSCC cases.

**Methods:**

The retrospective study was carried out on 35 formalin-fixed paraffin sections of nodal metastasizing OSCC cases selected from the Oncology Centre, Faculty of Medicine, Mansoura University archives from 2018 to 2023. Immunohistochemistry for Drp1 and cortactin was done. The immune reactivity of both proteins was evaluated using computer-assisted digital image analysis. Statistical analysis was performed to identify significant differences and correlations between both markers in tissues associated with progressing OSCC cases using Chi-Square, Monte Carlo, One-Way ANOVA, and Spearman tests. The p-value less than 0.05 was considered statistically significant.

**Results:**

Drp1 expression was statistically significant to grades of primary OSCC (*p* = 0.015), while insignificant to grades of epithelial dysplasia (*p* = 0.123) and metastatic lymph nodes (LNs) (*p* = 0.212). Statistically significant differences between dysplastic epithelium & primary tumor, dysplastic epithelium & metastatic LNs, and primary tumor and metastatic LNs were observed (*p* values were 0.014, 0.001, 0.034, respectively). On the other hand, Cortactin expression revealed no statistically significant differences across the three groups. However, statistically significant differences between dysplastic epithelium & primary tumor, dysplastic epithelium & metastatic LNs, and primary tumor and metastatic LNs were found (*p* values were 0.014, 0.001, 0.034, respectively). Moreover, the Spearman test presented a strong positive correlation between Drp1 and cortactin expression in the studied cases.

**Conclusion:**

Expressions of both Drp1 and cortactin relatively explain their great role in the propagation and the carcinogenesis of OSCC.

## Background

Oral squamous cell carcinoma (OSCC) is the most common oral cancer worldwide that originates from the squamous cells of the oral cavity [1]. It has high rates of morbidity and mortality, primarily due to late diagnosis, early LN metastasis, recurrence, and treatment failure [1, 2].

Various biological changes are known to pave the way for OSCC development. OSCC begins with epithelial dysplasia, a precursor condition which is often the first stage of carcinogenesis characterized by the distortion of epithelial cellular uniformity and architectural structure [3, 4, 5]. Thence, the epithelial cells penetrate the Basement membrane (BM) expanding into the underlying submucosal tissue, where the extracellular matrix (ECM) encloses muscle, bone, and fat [3]. In the ECM, the cancer cells migrate from the primary site, enter the vascular system, and reach a secondary site (nodal and or distantly) to other organs [6, 7].

Despite significant improvements in diagnostic and treatment techniques, the OSCC’s 5-year survival rate remains unchanged [8]. This might be attributed to the poorly understood molecular and cellular mechanisms underlying the pathogenesis, which remain a subject of paramount importance [9, 10]. For the dysplastic epithelial transformation to occur and for the propagating nodal and distant metastasis, tumor cells need motility. This dynamic activity requires energy primarily obtained from the mitochondria, the main source of the adenosine triphosphate “ATP” production [11].

Mitochondria continuously adjust their number, shape, and function based on the cell's needs, primarily through two processes: fission (splitting) and fusion (merging). These processes are key mechanisms in mitochondrial dynamics [12]. Each cell maintains a delicate balance between mitochondrial fusion and fission to ensure proper mitochondrial function. Disruptions in this balance have been shown to play a crucial role in the initiation and progression of tumors [13, 14].

Dynamin-related protein1 (Drp1) is a key protein involved in mitochondrial fission; it exists as a cytosolic protein that actively needs to translocate to the mitochondrial outer membrane to promote mitochondrial fission [12, 15]. Up-regulation of Drp1 was linked to metabolic reprogramming, resulting in disease progression through enhanced migration, invasion, and metastatic potential in cancers such as pancreatic [16], and esophageal squamous cell carcinoma [17]. Although the role of Drp1 in mitochondrial division is well-studied, its biological effects in OSCC are not fully understood. The available studies reported Drp1 overexpression in OSCCs suggesting that enhanced mitochondrial fission provides the daughter mitochondria needed for the rapid proliferation of OSCC cells, and increased total level of ATP, resulting in high invasiveness [18, 19]. Meanwhile, it was reported that OSCC patients with low Drp1 expressions had better overall survival than those with high Drp1 levels, confirming that loss of Drp1protein in OSCC causes mitochondrial elongation with subsequent inhibition of cell proliferation [19], [20].

Besides the large amount of ATP obtained from the mitochondria, the essential prerequisite for cancer cell motility to metastasize is the dramatic reorganization of their actin cytoskeleton, which is crucial for maintaining the cell shape [21]. The actin cytoskeleton is the primary force-generating machinery in the cell that can produce pushing (protrusive) forces resulting in a structure termed invadopodia that exhibits proteolytic activity through promoting secretion of matrix metalloproteinases (MMPs) contributing to the penetration of the BM and metastasis [22, 23, 24]. Structurally, they are composed of an actin-rich core that includes actin activators and regulators, including the cortactin protein [25].

Cortactin is an actin-binding protein that promotes actin polymerization and rearrangement, playing a crucial role in invadopodia dynamics [26, 27]. Higher levels of cortactin were reported to be associated with higher histological grades, worse prognosis, and LN metastasis in OSCC studies [28, 29]. Moreover, the possible participation of cortactin in SCC carcinogenesis had been postulated in the early stages of OSCC, as its expression was significantly elevated in potentially malignant oral lesions, with higher levels observed in lesions with greater epithelial dysplasia [30].

The crosstalk between the mitochondria and the actin cytoskeleton is a highly coordinated bidirectional communication process that shapes the dynamics of each to regulate many cellular processes, including cell migration [31]. Substantial evidence obtained from studied breast, ovarian cancers, and hepatocellular carcinoma showed that invadopodia formation is one of the key steps in cell migration, and high energy production is required for the actin filaments assembly at the cell’s leading edge. To accommodate this demand, the mitochondrial fission event, which results in a large amount of ATP, is necessary for the redistribution and movement of mitochondria to the invadopodia region of the cells, where the energy demand is higher, to power cell migration [11, 32, 33, 34]. However, the interplay between the mitochondrial dynamics protein “Drp1” and the invadopodia-related protein “cortactin” in OSCC remains elusive. Therefore, it is hypothesized that the IHC study of Drp1 and cortactin proteins might throw a beam of light on their possible interplay in the carcinogenesis of OSCC.

## Material and methods

### Patients’ selection and data retrieval

The present retrospective study was carried out on 35 nodal metastasizing OSCC cases selected from the archives of the Oncology Centre, Faculty of Medicine, Mansoura University from 2018 to 2023. Two paraffin blocks were retrieved from each case, forming two distinct groups: Group 1 (primary tumors with dysplastic margins) and Group 2 (metastatic LN infiltration). Based on the WHO classification system for oral epithelial dysplasia (OED) [35], the study sample encountered 7 cases of mild dysplasia, 23 cases of moderate dysplasia, and 5 cases of severe dysplasia. According to the WHO grading system of OSCC [36], the majority of cases were moderately differentiated (22 cases), followed by poorly differentiated (9 cases), and the least common was well-differentiated OSCC (4 cases).

### Immunohistochemistry

Two sections from the formalin-fixed paraffin-embedded blocks were cut at 4 μm thickness for IHC staining of Drp1 and cortactin proteins. Both markers were rabbit polyclonal antibodies obtained in a ready to use form. The sections were mounted on electrically charged Opti plus slides to ensure tissue adhesion. Immunostaining was performed using a standardized Avidin–Biotin complex (ABC) method.

The IHC procedure involved several steps: first, deparaffinization and rehydration in descending grades of alcohol. Peroxide quenching with 3% hydrogen peroxide. Antigen retrieval was performed using citrate buffer solution (PH = 6), followed by primary antibody incubation with Drp1 and cortactin for 60 min in a humidity chamber. After washing, slides were incubated with a secondary antibody and streptavidin peroxidase at room temperature. The chromogenic reaction was developed using Diamine benzidine tetra-hydrochloride “DAB” counterstained with Harris hematoxylin and dehydrated before mounting with fluoro-mount G. This process allowed effective visualization of Drp1 and cortactin expression in OSCC tissues. The positive controls were sections of brain and esophagus for Drp1 and cortactin antibodies respectively. The negative controls obtained by replacement of the primary antibodies by plain phosphate buffer solution (PBS) to assess the background staining.

### Assessment of IHC results

Slides were photographed using an Olympus® digital camera installed on an Olympus® microscope with a 1/2 X photo adaptor, using a 40X objective in the faculty of Dentistry, Mansoura University. Four randomly selected positive fields were taken from each slide. The resulting image was analyzed on an Intel® core I7® based computer using Fiji ImageJ (version 1.51r; NIH, Maryland, USA) software. Sections of the examined cases were evaluated based on the intensity and percentage area of positive cell staining for Drp1 and cortactin antibodies. A “staining intensity quantification protocol” was used to assess the staining intensity. This protocol measures the means of gray value within the selection, the pixel intensity values for any color in ImageJ range from 0 to 255, wherein 0 represents the darkest shade and 255 represents the lightest shade of the color [37]. The regions of interest (ROI) were selected to represent the positive reaction, and the staining intensity was measured as the “mean gray value “parameter. The average staining intensities for all measured ROI from four fields of vision were calculated for each sample, and the measured data were exported to an Excel sheet. Based on the positive and negative control tissue specimens analysis, the intensity of the samples for both markers was scored on a scale from 0 to 3 as follows: 0 (negative), 1 (weak), 2 (moderate), and 3 (strong).

The percentage area of Positive Cells was measured in the form of an area percent per four fields using a magnification of 400 by light microscopy transferred to the monitor. Images were converted into 8-bit types of grayscales and then masked by red binary color to adjust the threshold and highlight the required area to measure. The percentage area of positive cells was scored for both markers, with Drp1 being categorized as follows: 0 (0% of tumor cells), 1 (< 10%), 2 (10–50%), 3 (50–75%), and 4 (> 75%) [38]. For cortactin, the scoring was: 0 (0%), 1 (< 10%), 2 (10–50%), 3 (50–80%), and 4 (> 80%) [39]. The final expression score for each marker was obtained by multiplying the intensity score by the percentage score of positive cells. Scores of Drp1 beyond 3 were considered positive expressions [38], and the results for cortactin were categorized as negative (0), mild (1–4), intermediate (6–8), and strong (9–12) [39].

### Statistical analysis

Statistical analysis of the data was done by using the Excel program and Statistical Package Social Sciences (SPSS) software program to assess Drp1 and cortactin IHC expressions in OSCC tissues, their significant differences, and potential correlations with tumor progression. Quantitative data were described using median or mean ± standard deviation, depending on the distribution. Qualitative data were expressed as numbers and percentages, using Chi-Square and Monte Carlo tests to compare between groups. One-way ANOVA and Kruskal–Wallis tests were used to compare multiple groups. Spearman correlation was used for continuous, non-normally distributed data. A *p*-value less than 0.05 was considered statistically significant.

## Results

### Clinicopathological characteristics

As shown in Table 1, the study sample involved 35 patients aged 26 to 82 years, with a mean age of 58.86. It included 18 males and 17 females, showing a slight male predominance. Most cases (48.6%) were seen in the tongue, followed by the buccal mucosa (14.3%). Most cases were diagnosed with stage IV (68.6%), followed by stage III (25.7%). According to the WHO grading system of OED, the study sample encountered three grades: mild (20%), moderate (65.7%), and severe (14.3%). According to the WHO grading system of OSCC, the majority of cases were moderately differentiated (62.9%), followed by poorly differentiated (25.7%), and the least common was well-differentiated OSCC (11.4%). In metastatic LNs, malignant squamous cells were observed to invade as epithelial pearls, large solid nests, or individual dispersed cells.

**Table 1:** Clinicopathological characteristics of the studied cases

Clinicopathological variables		Frequency	%
**Age/years**	** < 60**	19	54.3
	** ≥ 60**	16	45.7
**Patients’ sex**	**Male**	18	51.4
	**Female**	17	48.6
**Tumor Site**	**Tongue**	17	48.6
	**Buccal mucosa**	5	14.3
	**Retromolar area**	4	11.5
	**Alveolar mucosa**	2	5.7
	**Others** (Cheek- Gingiva- Hard palate-lip)	7	20.3
**TNM stage**	**I**	1	2.9
	**II**	1	2.9
	**III**	9	25.7
	**IV**	24	68.6
**Grade of OED**	**Mild**	7	20.0
	**Moderate**	23	65.7
	**Severe**	5	14.3
**Histologic grades of OSCC**	**Well-differentiated**	4	11.4
	**Moderately differentiated**	22	62.9
	**Poorly differentiated**	9	25.7

Frequency table.

### The IHC expression of Drp1 concerning the different clinicopathological variables

**Fig. 1:**
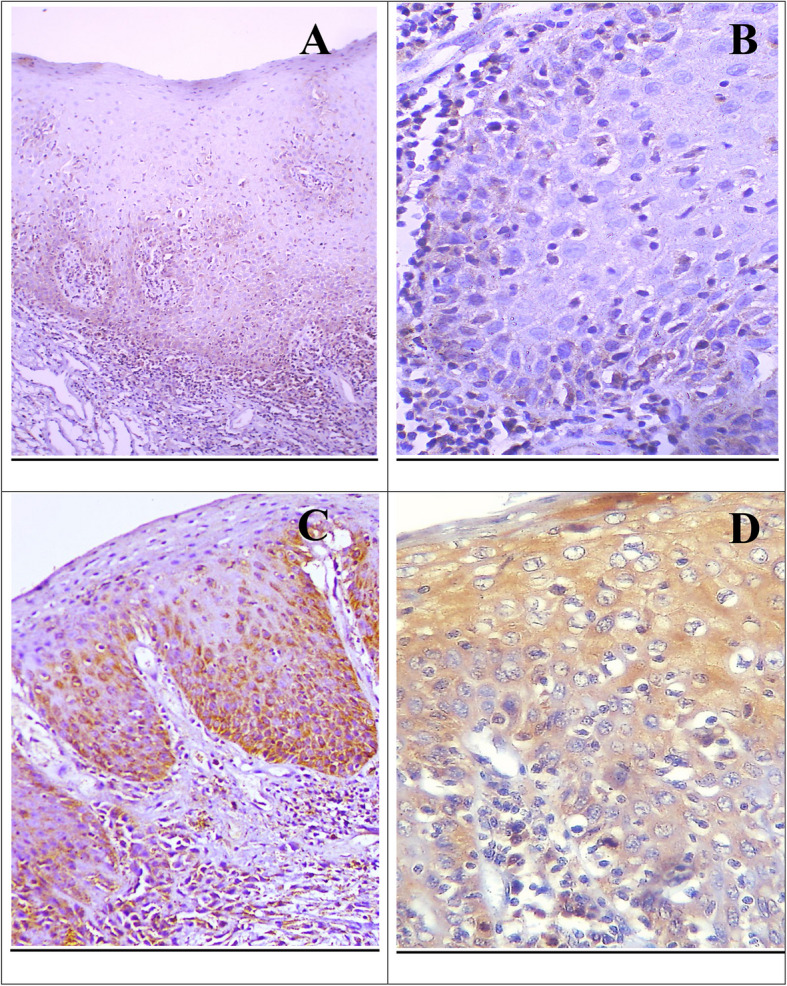
Weak Drp1 IHC expression in the basal and supra-basal cells of mild OED (**A**, **B**) (× 100, × 400), strong cytoplasmic and membranous reaction in moderate OED till half of the epithelial thickness (**C**) (× 200), and strong cytoplasmic reaction in severe OED (**D**) (× 400)

Concerning grades of OED, Drp1 expression was observed as a cytoplasmic and membranous reaction in the basal and supra-basal layers in mild dysplasia, extending to half the epithelial thickness in moderate dysplasia and throughout the entire epithelium in severe dysplasia (Fig. 1). Out of 35 cases, 22 showed positive immunoreactivity for Drp1, with the highest occurrence in moderate dysplasia (63.6%), followed by severe dysplasia (22.7%), and the lowest in mild dysplasia (13.6%). Using the Monte Carlo test, the differences in Drp1 expression across dysplasia grades were statistically insignificant (*p* = 0.123) (Table 2).

**Table 2:** Drp1 immunoreaction among different grades of OED

Dysplastic epithelium	**Total**	**%**	**Drp1**		**Test of significance**
	***N*** **= 35**		**Negative** ***N*** **= 13(%)**	**Positive** ***N*** **= 22(%)**	
Mild	7	20.0	4(30.8%)	3 (13.6%)	*p* = 0.123
Moderate	23	65.7	9(69.2%)	14 (63.6%)	
Severe	5	14.3	0	5 (22.7%)	

Used test: Monte Carlo test.

**Fig. 2:**
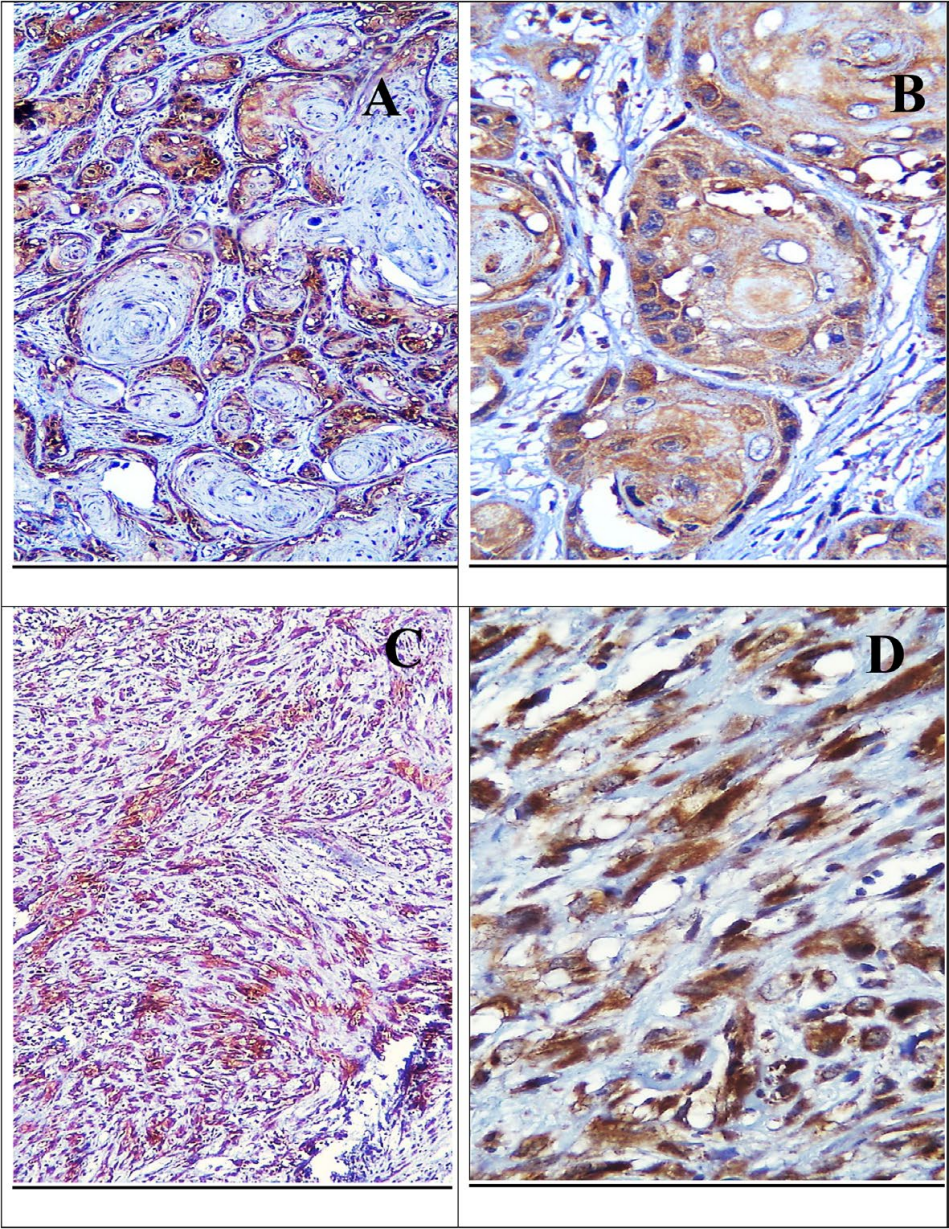
Strong cytoplasmic Drp1 IHC expression in epithelial pearls of well-differentiated OSCC (**A**) (× 100), epithelial nests of moderately differentiated OSCC (**B**) (× 400), and dispersed malignant epithelial cells of poorly differentiated OSCC (**C**, **D**) (× 100, × 400)

Regarding Drp1 IHC expression among different grades of OSCC, the expression appeared as a cytoplasmic reaction in both well-differentiated and moderately differentiated OSCC cases, while in poorly differentiated cases, the reaction was stronger in the cytoplasm of the dispersed malignant epithelial cells (Fig. 2). The positive immunoreaction was observed in 22 out of the 35 studied cases. The highest positivity was found in moderately differentiated OSCC (45.5%) and poorly differentiated OSCC (40.9%), while the lowest positivity was in well-differentiated OSCC (13.6%). The differences between the groups were statistically significant (*p* = 0.015), as determined by the Monte Carlo test (Table 3).

**Table 3:** Drp1 immunoreaction among different grades of OSCC

Primary tumor WHO	**Total**	%	**Drp1**		**Test of significance**
	***N*** **= 35**		**Negative** ***N*** **= 13(%)**	**Positive** ***N*** **= 22(%)**	
Well-differentiated	4	11.4	1 (7.7%)	3 (13.6%)	*p* = 0.015*
Moderate differentiated	22	62.9	12 (92.3%)	10 (45.5%)	
Poorly differentiated	9	25.7	0	9 (40.9%)	

Used test: Monte Carlo test.

**Fig. 3:**
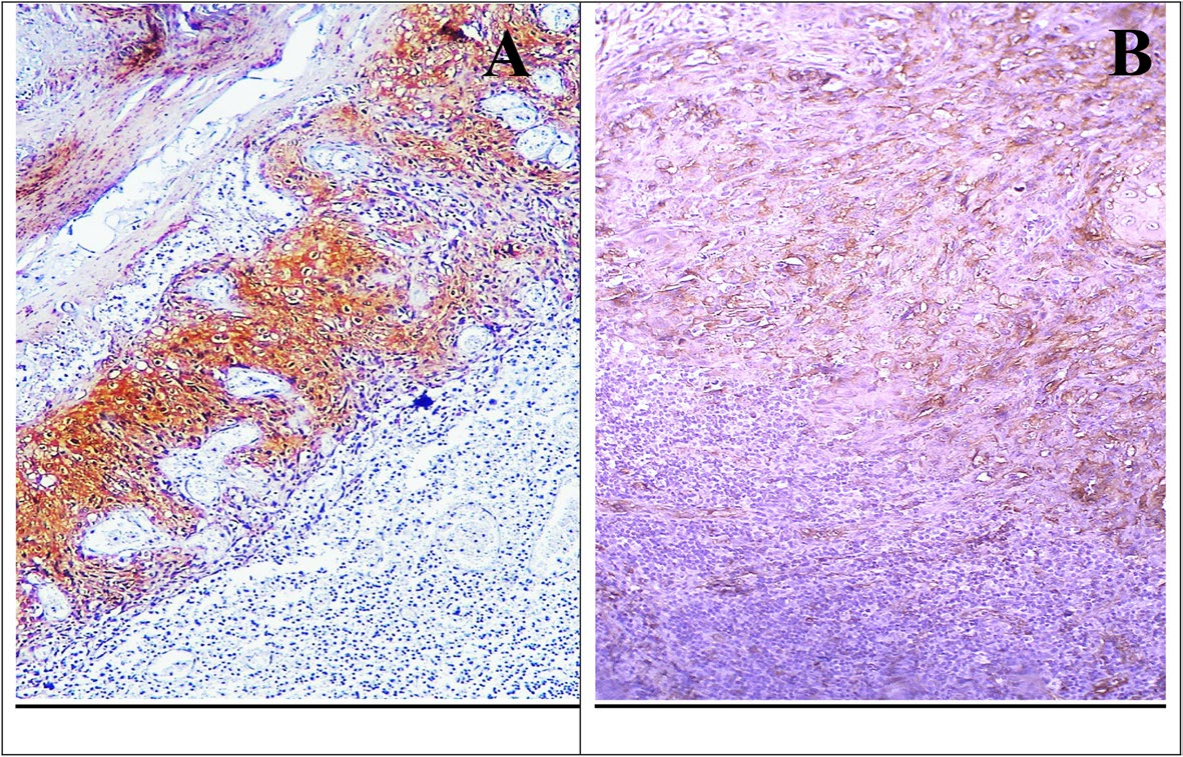
Metastatic LN showing strong cytoplasmic Drp1 expression in the epithelial nests (**A**), and moderate cytoplasmic Drp1 expression in dispersed epithelial cells (**B**) (× 100)

In metastatic LNs, Drp1 expression was observed high in the malignant cells invading LNs (Fig. 3). The mean ratio of infiltrated LN to total excised was 0.631 ± 0.515, with no statistical significance between groups (*p* = 0.212), using the Mann–Whitney U test (Table 4).

**Table 4:** Drp1 immunoreaction among metastatic LNs

	**Total**	**%**	**Drp1**		**Test of significance**
	***N*** **= 35**		**Negative** ***N*** **= 13(%)**	**Positive** ***N*** **= 22(%)**	
**Ratio of infiltrated**	Mean ± SD		0.971 ± 0.694	0.631 ± 0.515	*p* = 0.212
**LN/total excised**	Median (min–max)		0.875 (0.18–2.33)	0.488 (0.17–2.33)	

Used test: Mann Whitney U test.

Drp1 expression varied across the three groups; 45.7% of dysplastic epithelium cases showed positive Drp1 immunoreaction, while 54.3% were negative. In the primary tumor, 62.9% were positive, and 37.1% were negative for Drp1. In metastatic LNs, 80% showed positive Drp1 immunoreactivity, with only 20% negative. The One-way ANOVA test revealed statistically significant differences between dysplastic epithelium & primary tumor, dysplastic epithelium & metastatic LNs, and primary tumor and metastatic LNs (*p* values were 0.014, 0.001, 0.034, respectively) (Table 5).

**Table 5:** Drp1 immunoreaction between different studied groups

Drp1	Dysplastic epithelium		Primary tumor		Metastatic LNs	
	*N* = 35	%	*N* = 35	%	*N* = 35	%
**-VE**	19	54.3	13	37.1	7	20.0
** + VE**	16	45.7	22	62.9	28	80.0
	*p1* = 0.014*, *p2* = 0.001*, *p3* = 0.034*					

*Used test: One-way ANOVA test.*

*p1:* The difference between dysplastic epithelium & primary tumor.

*p2:* The difference between dysplastic epithelium & metastatic LNs.

*p3:* The difference between primary tumor and metastatic LNs.

On the other hand, Chi-square and Monte Carlo tests revealed no statistically significant differences concerning the following clinical variables; patient age (*p* = 0.172), sex (*p* = 0.826), tumor site (*p* = 0.270), and the TNM stage (*p* = 0.694).

### The IHC expression of cortactin concerning the different clinicopathological variables

**Fig. 4:**
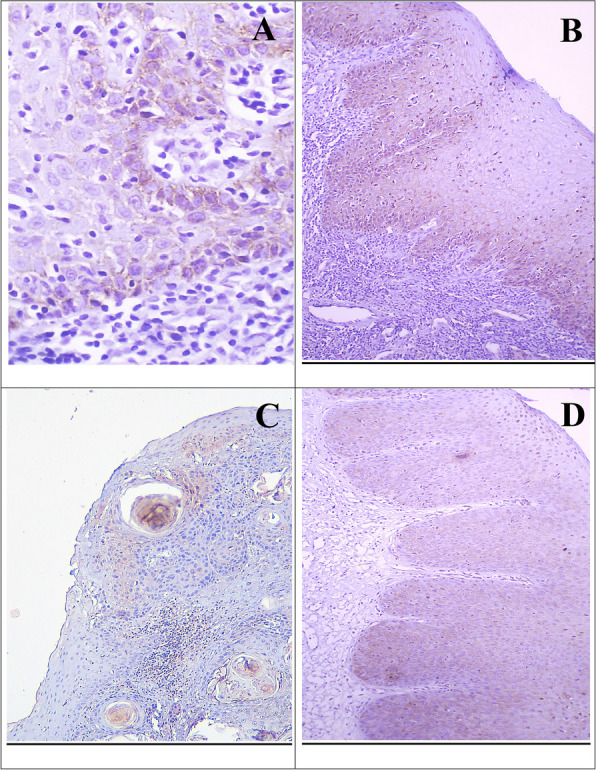
Weak cortactin IHC expression in the basal and supra-basal cells of mild OED (**A**) (× 400), extended to half the epithelial thickness in moderate OED (**B**) (× 100), and passing the midpoint of the thickness in severe OED (**C**, **D**) (× 100)

Cortactin expression in dysplastic epithelium showed a cytoplasmic and membranous pattern. In mild dysplasia, it was observed in the basal and supra-basal layers; in moderate dysplasia, it extended to half the epithelial thickness; and in severe dysplasia, it covered the entire epithelium (Fig. 4). Positive immunoreactivity was observed in all dysplastic cases (100%), with generally weak reaction scores distributed as; 25% in mild, 62.5% in moderate, and 12.5% in severe dysplasia. Statistical analysis, using the Monte Carlo test, revealed no significant difference between the grades (*p* = 0.883) (Table 6).

**Table 6:** Cortactin immunoreaction among different grades of dysplasia

Dysplastic epithelium	**Cortactin**			**Test of significance**
	**Weak**	**Moderate**	**Strong**	
Mild	4 (25%)	2 (20%)	1 (11.1%)	*p* = 0.883
Moderate	10 (62.5%)	7 (70%)	6 (66.7%)	
Severe	2 (12.5%)	1 (10%)	2 (22.2%)	

Used test: Monte Carlo test.

**Fig. 5:**
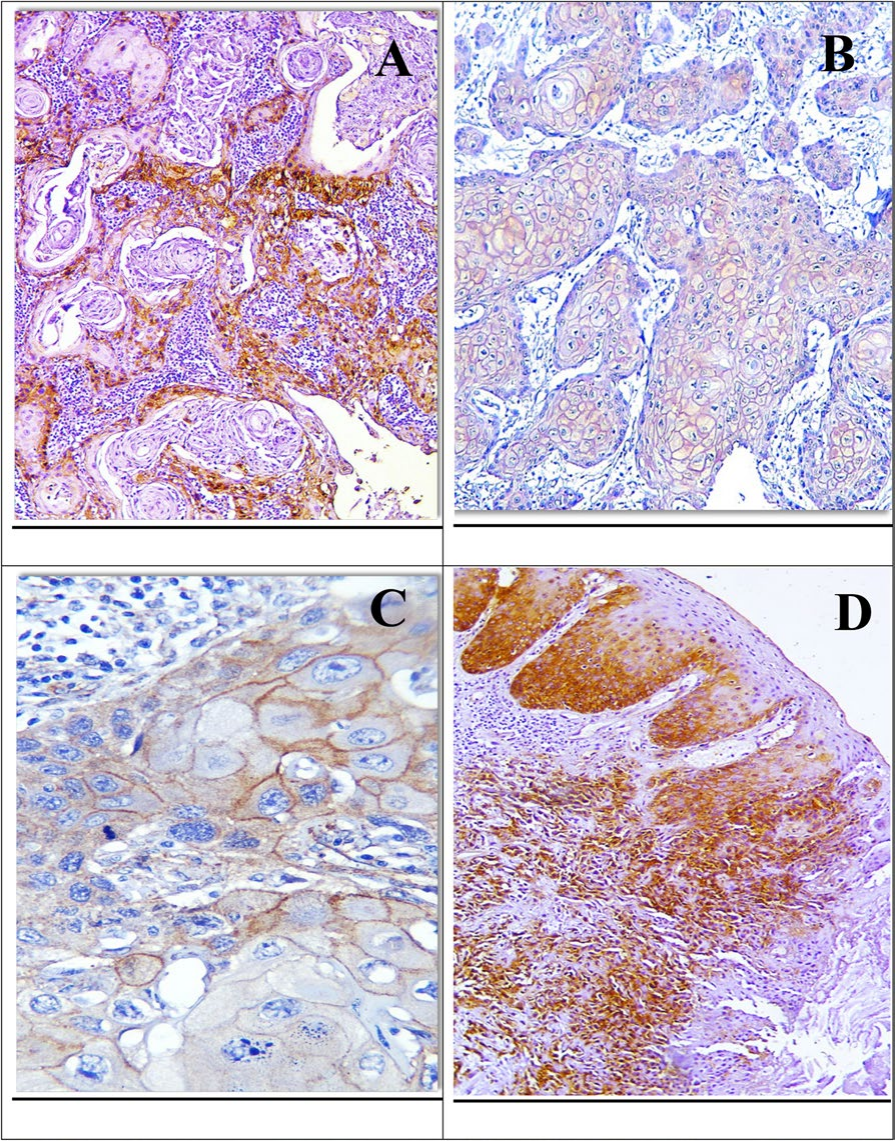
Cortactin IHC expression among OSCC grades; strong cytoplasmic in epithelial pearls of well-differentiated OSCC (**A**) (× 100), moderate membranous expression in epithelial nests of moderately differentiated OSCC (**B**, **C**) (× 100, × 400), and strong cytoplasmic in scattered malignant cells of poorly differentiated OSCC (**D**) (× 100)

Concerning cortactin IHC expression among grades of primary OSCC, the expression varied by grade’s differentiation; it was noticed cytoplasmic in well-differentiated and poorly differentiated cases, cytoplasmic and membranous in moderately differentiated cases (Fig. 5). All cases involved were positive, with generally weak staining observed in 6.2% (one case) of well-differentiated, 75% (12 cases) of moderately differentiated, and 18.8% (3 cases) of poorly differentiated OSCC. Monte Carlo test revealed no significant differences between the grades (*p* = 0.599) (Table 7).

**Table 7:** Cortactin immunoreaction among different grades of primary OSCC

Primary tumor	Cortactin			Test of significance
	**Weak**	**Moderate**	**Strong**	
**Well-differentiated**	1 (6.2%)	1 (10.0%)	2 (22.2%)	*p* = 0.599
**Moderate differentiated**	12 (75.0%)	6 (60.0%)	4 (44.4%)	
**Poorly differentiated**	3 (18.8%)	3 (30.0%)	3 (33.3%)	

Used test: Monte Carlo test.

**Fig. 6:**
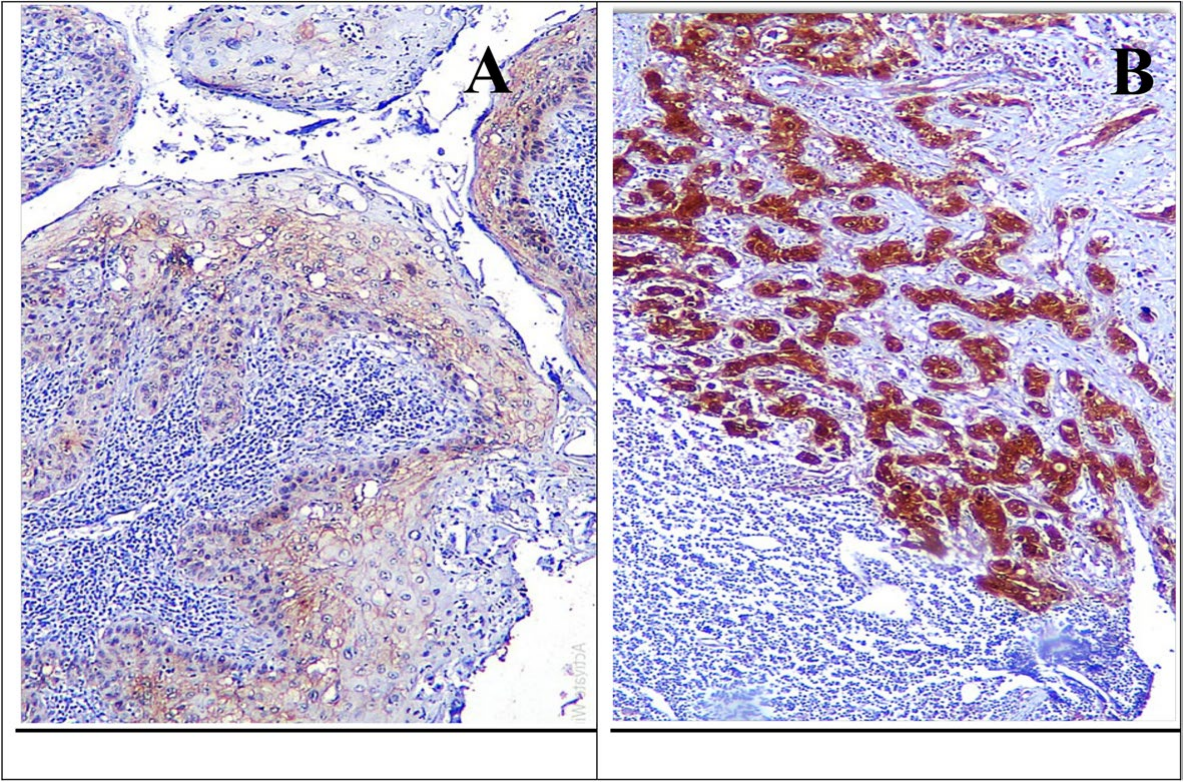
Cortactin expression in metastatic LN showing moderate reaction in the invading epithelial nests (**A**), and strong reaction in scattered groups of epithelial cells (**B**) (× 100)

In metastatic LNs, all cases showed positive cortactin staining, with malignant cells invading exhibiting moderate to strong IHC staining (Fig. 6). The mean ratio of infiltrated LN to total excised was 0.895 ± 0.488. Kruskal–Wallis test revealed statistical insignificance between the groups (*p* = 0.517) (Table 8).

**Table 8:** Cortactin immunoreaction among metastatic LNs

	**Cortactin**			**Test of significance**
	**Weak**	**Moderate**	**Strong**	
**Ratio of infiltrated**	0.775 ± 0.63	0.895 ± 0.488	0.575 ± 0.675	*p* = 0.517
**LN/Total excised**	0.488 (0.18–2.33)	0.78 (0.37–1.75)	0.318 (0.17–2.33)	

Used test: Kruskal Wallis test.

Cortactin expression varied among the three groups; in the dysplastic epithelium, 26 cases (74.3%) showed weak expression, and seven cases (20%) had a moderate reaction, with no strong expression observed. In primary tumors, 16 cases (45.7%) showed weak expression, 10 cases (28.6%) were moderate, and nine cases (25.7%) were strong. In metastatic LNs, most cases (16 cases, 45.7%) exhibited strong expression. The One-way ANOVA test revealed statistically significant differences between dysplastic epithelium & primary tumor, dysplastic epithelium & metastatic LNs, and primary tumor and metastatic LNs (*p* value were 0.001, 0.001, 0.005, respectively) (Table 9).

**Table 9:** Cortactin immunoreaction between different studied groups

Cortactin	Dysplastic epithelium		Primary tumor		Metastatic LN	
	N = 35	%	*N* = 35	%	*N* = 35	%
**Weak**	26	74.3	16	45.7	10	28.6
**Moderate**	7	20.0	10	28.6	9	25.7
**Strong**	2	5.7	9	25.7	16	45.7
	*p*1 = 0.001*, *p*2 = 0.001*, *p*3 = 0.005*					

Used test: One-way ANOVA test.

*p*1: The difference between dysplastic epithelium & primary tumor.

*p*2: The difference between dysplastic epithelium & metastatic LNs.

*p*3: The difference between primary tumor and metastatic LNs.

On the other hand, Chi square and Monte Carlo tests revealed no statistically significant differences concerning the following clinical variables; patient age (*p* = 0.776), sex (*p* = 0.785), tumor site (*p* = 0.212), and the TNM stage (*p* = 0.477).

nstrated similar findings across the different histologi

### Correlation between Drp1 and cortactin expressions

**Fig. 7:**
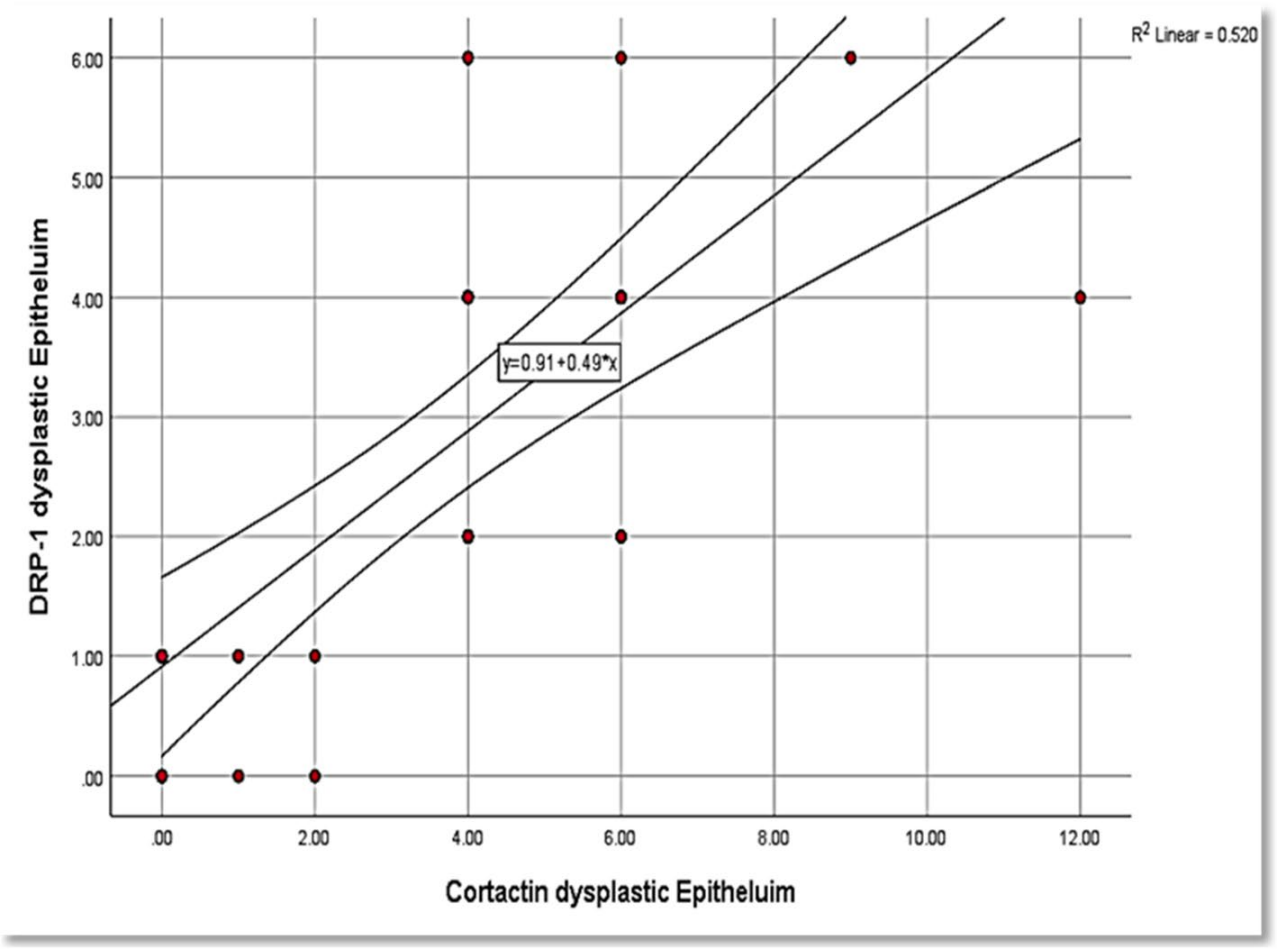
Correlation between cortactin expression and Drp1 expression in the dysplastic epithelium among the studied cases

**Fig. 8:**
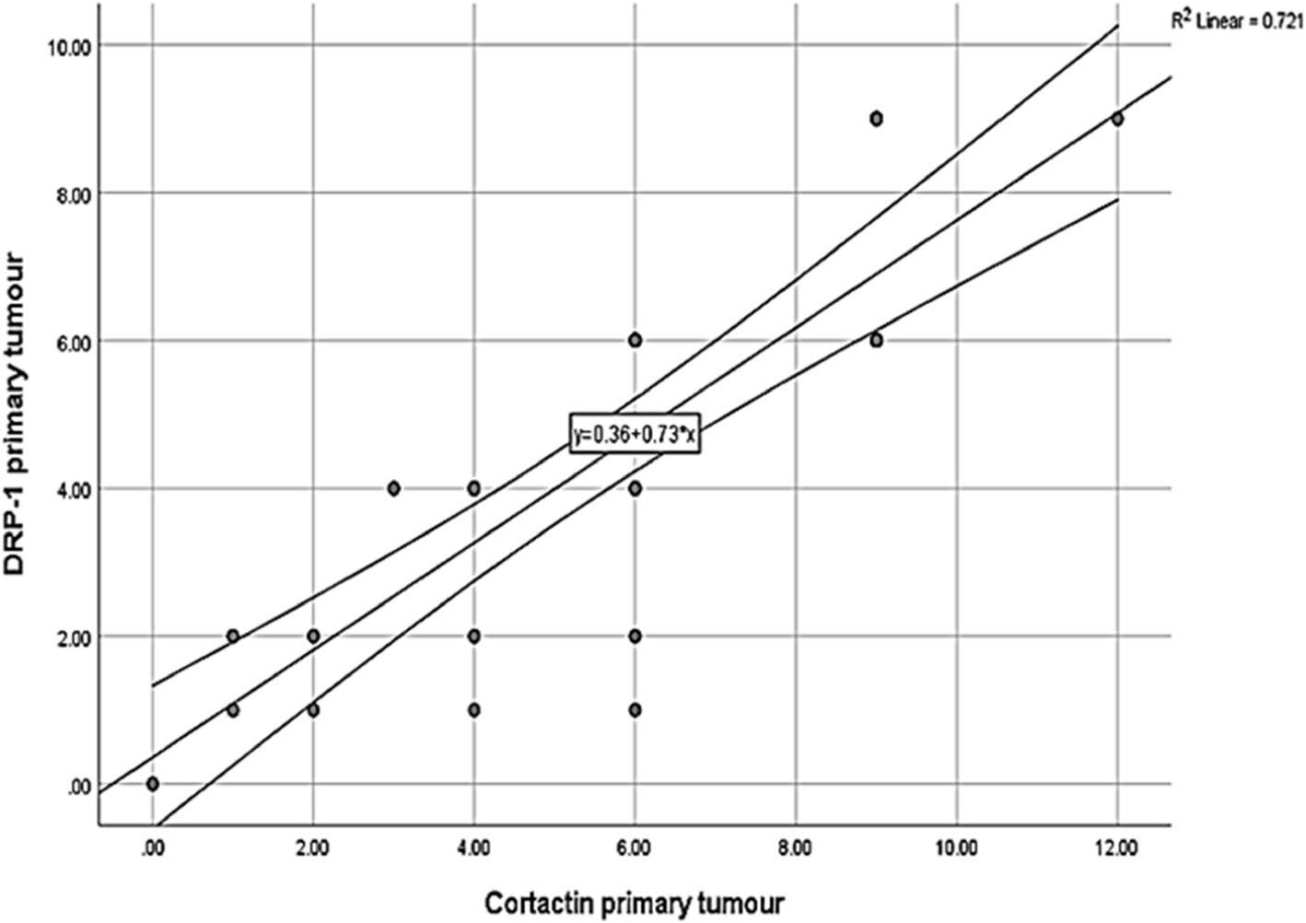
Correlation between cortactin expression and Drp1 expression in primary tumor among the studied cases

**Fig. 9:**
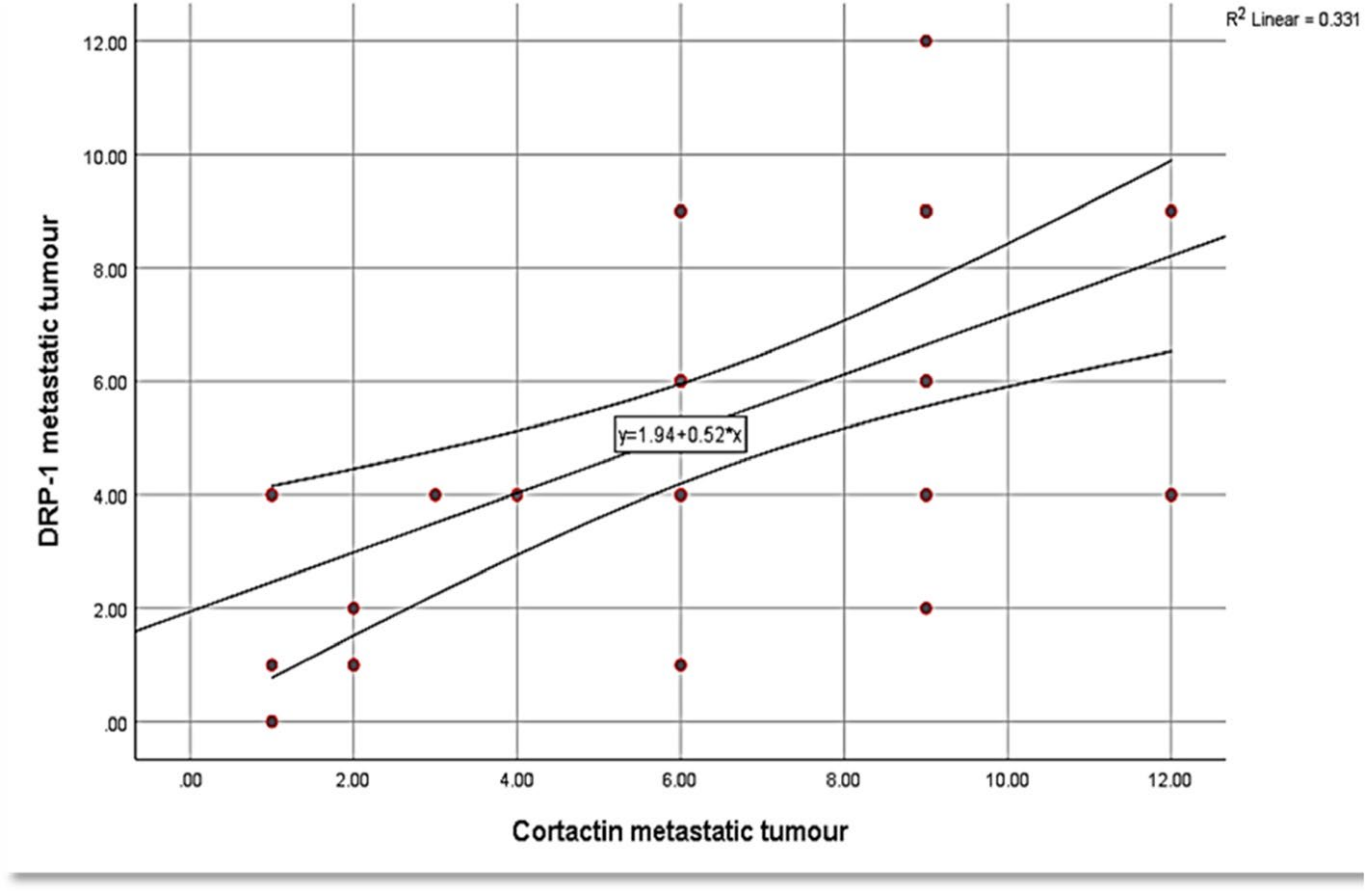
Correlation between cortactin expression and Drp1 expression in tumor infiltrating LNs among the studied cases

Relatively, both markers demonstrated similar findings across the different histologic groups of OSCC. Spearman test presented a strong positive correlation between Drp1 and cortactin expressions in the studied cases (Table 10) (Figs. 7, 8, 9).

**Table 10:** Correlation between Drp1 and cortactin expressions in the studied OSCC cases

	**r**	***p* value**
Dysplastic epithelium	0.782	0.001*
Primary tumor	0.841	0.001*
Metastatic LNs	0.564	0.001*

Used tests: Spearman test.

## Discussion

Oral squamous cell carcinoma is the most common malignant tumor among head and neck cancers, with an unfavorable prognosis [27]. Its development involves complex and multifactorial processes influenced by genetic alterations, epigenetic changes, and tumor microenvironment disturbance, eventually leading to invasive tumor [40, 41]. A thorough understanding of the pathological etiology behind OSCC progression is essential for developing effective therapeutic strategies [20]. For dysplastic transformation and metastasis to occur, tumor cells require motility, and this dynamic activity depends on energy and phenotypic changes in cellular organelles, particularly the mitochondria [11].

Mitochondria are highly dynamic organelles that constantly undergo fusion and fission to form an active network in response to cellular stimuli [42]. While increased mitochondrial fission is a known phenomenon in many cancers to meet energy demands, its role in oral squamous cells as a potential indicator of OSCC progression remains underexplored [19]. Drp1 is the key protein that regulates mitochondrial fission by being recruited from the cytosol to the outer mitochondrial membrane. However, its role in malignant tumor development and pathogenesis are still being studied [43].

Throughout the examination of the OED in the currently studied cases, Drp1 IHC expression was observed in most cases, particularly among moderate and severe dysplasia. The positive staining was found in the basal, parabasal, and spinous layers of the dysplastic epithelium. These findings were aligned with Ghosh et al. who observed increased Drp1 expression in oral dysplastic tissue, which may be attributed to mitochondrial dysfunction and structural fragmentation [19]. Similar results had been reported in other cancers, including esophageal SCC [44], skin cancer [45], and colorectal precancerous lesions [46], suggesting that Drp1 could serve as a biomarker for cancer progression.

Concerning Drp1 expression in grades of primary OSCC in the present study, Drp1 expression was positive and strong in poorly differentiated OSCC, while the well-differentiated group showed the least positive expression, with statistical significance between the groups. These results are consistent with previous studies where higher Drp1 levels were in OSCC tissues compared to normal tissues, and that patients with lower Drp1 expression had better prognoses [19, 20]. While similar findings were observed in cutaneous SCC, no statistical significance was found [47]. The possible reason for this enhanced mitochondrial fission in cancer cells might be attributed to the requirements for maintaining cellular homeostasis, proliferation rate, and evading apoptosis. However, its role as a potential indicator of OSCC progression is not well studied [19, 48]. Contrary to our findings, Zhai et al. reported that lower Drp1 expression in esophageal SCC was associated with higher invasiveness [44]. Moreover, decreased Drp1 expression had also been associated with advanced stages in colon and lung cancers, suggesting that Drp1 loss may contribute to tumor progression by causing mitochondrial dysfunction [49]. Although these findings appear contradictory, they highlight the varying roles of mitochondria in tumorigenesis across different cancers, emphasizing the need for a careful approach when studying Drp1's role in human cancer.

Regarding Drp1 expression in metastatic LNs, our studied cases revealed high expression, aligning with Kitamura and his coworkers, who observed higher Drp1 levels in metastatic cutaneous SCC than in the non-metastatic groups [47]. Additionally, a recent study on head and neck cancer found that high Drp1 expression was linked to increased cell motility and metastatic characteristics, suggesting Drp1 as a potential prognostic marker and target for therapy in head and neck cancer patients [38]. Furthermore, Drp1 upregulation had been associated with increased metastatic capacity in breast cancer and hepatocellular carcinoma, as metastatic cancer cells with higher Drp1 expression exhibited more fragmented mitochondria compared to non-metastatic cells [32, 50]. Overall, Drp1 protein levels were higher in tumorigenic and metastatic patient samples compared to non-metastatic tissues, suggesting that mitochondrial fission alters the metabolic programs of cancer cells to enhance their metastatic potential [51].

In the current study, Drp1 expression did not show any statistically significant correlation with clinical parameters such as age, sex, tumor site, or TNM stage, which was in parallel with previous studies [44, 52]. However, Kim et al. found a correlation between decreased Drp1 expression and gender, with lower levels more commonly observed in males in both lung and colon cancers, suggesting that genetic and physiological factors contributing to gender differences in cancer may help explain Drp1 expression variations [49].

In addition to the energy provided by mitochondria, tumor invasion and metastasis are achieved by the rearrangement of the actin cytoskeleton in the direction of cell movement, a phenomenon known as invadopodia which are actin-rich protrusions that enhance the proteolytic activity in invasive carcinoma [39]. Cortactin, a cytoskeletal protein, plays a key role in stabilizing and organizing branched actin networks by promoting polymerization and the assembly of actin monomers [53].

Cortactin-positive immunoreactivity was observed in all studied cases of OED, with varying distribution and intensity. However, the positivity was generally weak, appeared as cytoplasmic and membranous reactions in the basal, parabasal, and spinous cell layers of the dysplastic epithelium, with no significant differences between groups. These findings were somewhat aligned with de Vicente et al., who also observed cytoplasmic and membranous cortactin expression at early stages of oral dysplasia. However, in their study, the frequency of positivity increased with the grade of dysplasia, suggesting a potential role of cortactin in the pathogenesis and progression of OSCC [30].

Concerning cortactin expression in different histological grades of OSCC, the expression was observed in all cases of OSCC, showing cytoplasmic and membranous reactions. The immunostaining was generally weak across different histological grades, with no significant differences. The association between cortactin expression and tumor differentiation is not fully understood, as previous studies have shown both positive and negative associations. For instance, Sengüven Toközlü et al. found no significant link between cortactin expression and tumor differentiation, similar to the findings of the present study [53]. However, Mitre and his coworkers reported higher cortactin expression in well-differentiated tumors compared to moderately and poorly differentiated ones [27], while Hofman et al. found cortactin overexpression associated with higher histologic grade [54]. These conflict results highlight the genetic diversity of OSCC, indicating that further investigations are needed to clarify the role of cortactin in OSCC prognosis.

Regarding cortactin expression in metastatic LNs in the current study, the expression was moderately and strongly stained in the malignant cells invading LNs. This observation was compatible with previous studies reported that cortactin overexpression had been frequently correlated with parameters that imply a worse prognosis in OSCC, including LN involvement, suggesting this protein is a prognostic marker for invasive and metastatic OSCC [27], [28]. Mohammed et al. explained that increased cortactin expression may reflect the need for neoplastic cells to maintain a stable intracellular actin assembly to facilitate their spread from the primary tumor site to distant locations [39].

The current study revealed a statistically significant difference in cortactin expression across dysplasia, primary tumors, and metastatic LNs. Limited data exists on this correlation; Rodrigo et al. found a strong positive correlation between cortactin expression in premalignant lesions and invasive tumors [55]. Similarly, a recent study by Mitre et al. reported cortactin overexpression in OSCC compared to healthy oral mucosa, suggesting that active invadopodia in OSCC may contribute to its unfavorable prognosis [27].

Furthermore, the present study found no statistically significant association between cortactin expression and clinical parameters such as age, sex, tumor site, or TNM stage which aligns with the findings of Hofman et al. [54] and Sengüven Toközlü et al. [53]. Additionally, Mitre et al. reported no correlation between cortactin overexpression and the clinical stage [27]. However, other studies showed that high cortactin expression was significantly associated with larger tumor size [29, 53] and higher TNM stage [54].

A strong positive correlation in the present study was found between Drp1 and cortactin expressions in different histologic groups of OSCC. This aligns with the well-established association between the actin cytoskeleton and mitochondria. Yadav et al. [31] suggests two mechanisms for how mitochondria influence actin cytoskeletal dynamics and induce invadopodia formation. The first mechanism, actin polymerization is an energy-demanding process, thus mitochondria provide ATP for the process. Second, proteins regulating mitochondrial dynamics, such as Drp1, can affect actin organization, so, in the absence of Drp1, mitochondria fail to divide properly and cannot be efficiently trafficked to areas where active actin remodeling occurs.

On the other hand, recent studies had shown that the cytoskeleton, particularly actin polymerization, plays a crucial role in regulating mitochondrial dynamics, positioning, and function. Actin aids Drp1 in the pre-constriction process of mitochondrial fission by helping Drp1 oligomers form ring-like structures around the outer mitochondrial membrane, leading to mitochondrial constriction and eventual division. Since mitochondrial circumferences are often larger than Drp1 ring diameters, actin helps pre-constrict mitochondria at mitochondria-endoplasmic reticulum contacts, reducing their diameter and facilitating Drp1-mediated scission [56, 57]. Another suggested mechanism for actin involvement in mitochondrial fission is the transient accumulation of actin and its binding protein, cortactin, on the outer mitochondrial membrane during the fission event. Thereby, suppression of cortactin expression inhibited mitochondrial fragmentation, indicating that cortactin plays a crucial role in maintaining mitochondrial dynamics [31, 58].

Although many therapies have been applied in OSCC, these therapies are still unsatisfactory. The role of the mitochondria and the actin cytoskeleton in OSCC therapy has recently attracted increasing attention, however, many mechanisms remain unclear. Bai et al. reported that suppression of mitochondrial fission may induce apoptosis in OSCC cells by releasing cytochrome c [59]. On the other hand, Ramos et al. suggested that blocking the cortactin oncogenic pathways and targeting genes amplified in chromosome band 11q13 may suppress tumor progression, and metastasis [60]. Therefore, there is a need for further research lines on key aspects of Drp1 and cortactin with a likely influence on oral carcinogenesis.

## Conclusion

Drp1 and cortactin play significant roles in malignant transformation and LN metastasis, highlighting their potential role as cancer biomarkers. Drp1 overexpression correlates with higher histologic grades of OED and OSCC, making it a potential prognostic marker for tumor aggressiveness and invasion. Moreover, cortactin overexpression was correlated with LN involvement, marking it as a metastasis predictor. However, the underlying molecular mechanisms concerning the interplay between the mitochondria and the actin cytoskeleton remain to be fully understood. Future IHC studies and cell lines should explore the potential link between both proteins in OED and OSCC, which may lead to better insights and therapeutic advancements.

## Data Availability

No datasets were generated or analysed during the current study.

## Abbreviations

OSCC: Oral squamous cell carcinoma
Drp1: Dynamin-related protein1
IHC: Immunohistochemistry
LNs: Lymph nodes
BM: Basement membrane
ECM: Extracellular matrix
ATP: Adenosine triphosphate
MMPs: Matrix metalloproteinases
OED: Oral epithelial dysplasia
ABC: Avidin–Biotin complex method
DAB: Diamine benzidine tetra-hydrochloride
PBS: Phosphate buffer solution
ROI: Regions of interest
SPSS: Statistical Package Social Sciences
